# An evaluation of uncertainty quantification methods and measures for deep learning outcome prediction models in head and neck cancer radiotherapy^☆^

**DOI:** 10.1016/j.phro.2026.100978

**Published:** 2026-04-26

**Authors:** Daniel C. MacRae, Luuk van der Hoek, Joëlle E. van Aalst, Suzanne P.M de Vette, Robert van der Wal, Hendrike Neh, Baoqiang Ma, Nanna M. Sijtsema, Matias A. Valdenegro-Toro, Peter M.A. van Ooijen, Lisanne V. van Dijk

**Affiliations:** a Department of Radiation Oncology, University Medical Center Groningen, University of Groningen, Groningen, the Netherlands; b Department of Artificial Intelligence, Bernoulli Institute, University of Groningen, the Netherlands

**Keywords:** Outcome prediction, Deep learning, Uncertainty quantification, NTCP, TCP

## Abstract

**Background and Purpose:**

Deep learning (DL) outcome prediction models show promise in radiotherapy but face limited clinical adoption due to concerns about prediction reliability. Although uncertainty quantification (UQ) can provide confidence estimates alongside predictions, there is currently little consensus on appropriate UQ approaches for DL-based outcome prediction. This study therefore evaluates and compares different UQ approaches for normal tissue complication probability (NTCP) and tumour control probability (TCP) models in head and neck cancer.

**Materials and Methods:**

Four published DL models were reproduced: two NTCP models predicting xerostomia and dysphagia at six months post-treatment, and two TCP models predicting two-year survival and locoregional control. Three UQ methods—Monte Carlo dropout, deep ensembles, and test-time augmentation—and three uncertainty measures—predictive entropy, variance, and mutual information—were evaluated. Models were retrained on development sets (NTCP: 964 patients, TCP: 255 patients), and assessed on independent validation sets (NTCP: 241 patients, TCP: 85 patients) using discriminative performance, calibration metrics, and sparsification analysis.

**Results:**

Incorporating UQ methods maintained comparable discriminative performance to baseline models across all endpoints (mean AUC: 0.72–0.73 vs 0.72). Deep ensembles and Monte Carlo dropout demonstrated strong calibration between uncertainty values and prediction accuracy, while test-time augmentation showed variable reliability. Entropy and variance consistently correlated with prediction accuracy, whereas mutual information proved unstable.

**Conclusions:**

Monte Carlo dropout and deep ensembles provide meaningful uncertainty estimates for NTCP and TCP prediction without compromising model performance. These methods show potential for selective prediction workflows where high-confidence predictions guide treatment decisions while uncertain cases are flagged.

## Highlights

• Uncertainty estimates help identify when NTCP/TCP model predictions are reliable.
• Uncertainty methods generally did not affect model performance (mean AUC: 0.72–0.73).
• Deep ensembles and Monte Carlo dropout had strong uncertainty-accuracy calibration.
• Test-time augmentation shows variable performance on multimodal outcome models.
• Mutual information proves unstable for binary outcome prediction tasks.

## Graphical abstract

**Figure:**
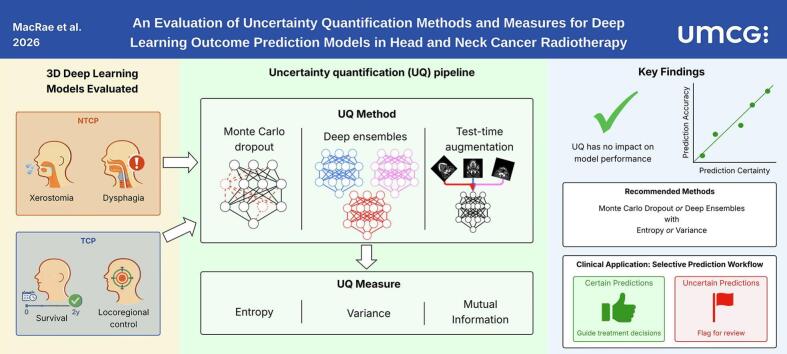

## 1 Introduction

Outcome prediction models can support radiotherapy by estimating patient-specific treatment responses. They integrate radiation dose distributions with patient and tumour characteristics to generate predicted probabilities of treatment outcomes [1]. These models are crucial for personalised treatment by identifying patient subgroups with distinct risk profiles, guiding the selection of patients for treatment strategies focused on dose reduction or treatment intensification. There are two main types of outcome prediction models in radiotherapy, normal tissue complication probability (NTCP) models estimate the risk of radiation-induced side effects, while tumour control probability (TCP) models predict treatment- or survival-related outcomes. In recent years, deep learning (DL) has emerged as a promising approach for predicting these outcomes. While conventional (logistic or Cox regression) models often face limitations when integrating a large number of features, such as multicollinearity, DL models can learn complex representations directly from 3D imaging, dose, and clinical data without these constraints [2]. Despite promising performance, the clinical adoption of DL outcome models in radiotherapy remains limited. Clinicians often hesitate to rely on these models because it is difficult to assess how a model arrives at a decision, whether this prediction is reliable, and how a model will behave for patients who differ from those in the training data [3].

**Fig. 1:**
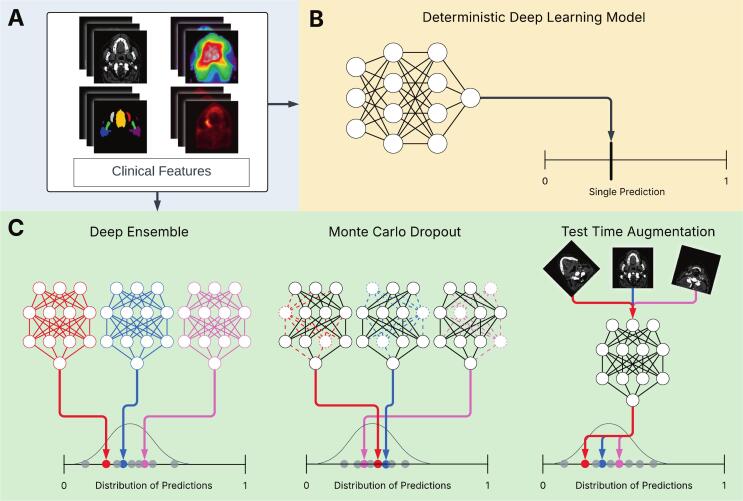
Schematic of data and models. A: The 3D (image) data and clinical features used in the TCP and NTCP DL models. B: A deterministic DL model, which represents how models are currently developed and serves as the baseline model for the current study. C: The same model, but implemented using three different uncertainty quantification techniques, which yields a distribution of predictions, rather than a single point prediction. Note that the NTCP DL models do not utilise the PET image, while the TCP DL models did not incorporate the 3D dose distribution.

Uncertainty quantification (UQ) could address some of these clinical adoption hurdles by providing estimates of model reliability alongside predictions. Rather than outputting just a single point prediction per patient, UQ represents predictions as probability distributions (Fig. 1). When these distributions are wider, the model is considered less certain about its prediction. These uncertainties arise from two principal sources. Aleatoric uncertainty (data uncertainty) reflects noise inherent to the data itself, such as variability in imaging or clinical assessments, and cannot be reduced by collecting more data. Meanwhile, epistemic uncertainty (model uncertainty) arises from limited knowledge of the model parameters and can be reduced with additional or more diverse training data [4]. Implementing UQ generally involves two components. First, an uncertainty method, such as Monte Carlo dropout [5] or deep ensembles [6], generates a set of predictions by introducing controlled randomness into the predictions. Second, an uncertainty measure, such as variance or entropy, quantifies how much those predictions vary—essentially summarizing the distribution into a single uncertainty value.

While UQ has been applied in radiotherapy tasks such as auto-contouring, its use in outcome prediction models is comparatively scarce [7]. Only a handful of studies have applied UQ to NTCP or TCP models, of which only three quantified prediction uncertainty [7]. They have applied diverse approaches—including dropout variational inference, test-time augmentation, conformal prediction, evidential DL, and Gaussian Process models—showing that predictions flagged as 'certain' are consistently more accurate and that rejecting uncertain predictions improves performance [8], [9], [10]. Collectively, these studies illustrate the promise of UQ for enhancing the reliability of DL outcome prediction models by flagging uncertain predictions. Nevertheless, they also expose substantial methodological heterogeneity and hence a lack of consensus on optimal methods, measures, and evaluation criteria for UQ in DL outcome prediction modelling; highlighting the need for comprehensive and systematic UQ evaluation.

The aim of this study is to evaluate the reliability of several UQ methods for DL–based radiotherapy outcome prediction models on a cohort of head and neck cancer (HNC) patients. By systematically comparing different UQ methods and measures using previously published NTCP and TCP models, we highlight their practical behaviour, strengths, and limitations in this context. The findings are intended to offer guidance and considerations for future work incorporating UQ into DL-based outcome modelling in radiotherapy.

## 2 Methods & materials

### 2.1 Deep learning models

Four previously-published DL models are used in this paper; two NTCP models, and two TCP models. For the NTCP models, the ResNet models for xerostomia, by Chu et al. [11], and dysphagia, by de Vette et al. [12], which both take 3D CT, dose distributions, and organ-of-interest contours, and clinical features as inputs, were retrained. The NTCP endpoints were assessed at six months post-radiation therapy. For grade 2–4 dysphagia, the physician-rated CTCAEv4.0 [13] rating was used and “moderate-to-severe” xerostomia corresponded to the two highest categories on the EORTC QLQ-H&N35 scale [14].

For the TCP models, the TransRP architectures from Ma et al. [15], [16], which utilise 3D CT, PET, gross tumour volume of primary tumours and pathological lymph nodes, and clinical features, were adapted to be compatible with the probabilistic UQ methods used in this work. As these UQ approaches require binary endpoints, the original event-based outcomes were binarised into 2-year survival and 2-year locoregional control (LRC), rather than the event-based endpoints in Ma et al. [16]. Accordingly, the final prediction layer was modified for binary outputs, and the loss function was changed to binary cross-entropy. Otherwise, all four models were kept consistent with those used in their respective original publications. Additional details regarding the models and the training procedure is outlined in Appendix A.

### 2.2 Patients and treatment characteristics

Two cohorts were used in the current study, maintaining similar inclusion criteria to the studies wherein the DL models were originally developed. Both cohorts consisted of HNC patients with squamous cell carcinoma who received (chemo)radiation therapy with curative intent at the University Medical Center Groningen. All patients were included in a prospective data registration program (NCT02435576). Patients were treated with 66–70 Gy to the primary tumour and an elective dose of 54.25 Gy, over the course of 6–7 weeks (33–35 fractions of 2 Gy), with or without concurrent platinum-based chemotherapy or cetuximab. Patients were excluded if they had previous surgery in the head and neck region (except for tonsillectomies or laser treatment of small glottic lesions).

The NTCP dataset consisted of patients treated between 2007 and 2024, for which both dysphagia and xerostomia toxicity scores were available at six months post-radiotherapy. This dataset is an extension of the original DL NTCP cohorts, which previously ran up to 2021. Exclusion criteria were previous treatment for HNC, induction chemotherapy, distant metastasis, age under 18, any fraction dose exceeding 2.4 Gy, not completing all planned treatment fractions, and missing any of the OAR contours which are used in the DL NTCP models. The NTCP cohort was randomly split into a development and independent validation set (80%/20%).

For the TCP models, the same cohort of oropharyngeal squamous cell carcinoma patients utilised by Ma et al. [15] are used. There, inclusion criteria were patients with histologically proven squamous cell carcinoma treated between 2010 and 2021, had only one primary tumour, and had both treatment planning contrast-CT and PET data available. Due to the binarization of the predicted endpoints, patients in the original (400 patient) cohort who had not reached the two years of follow-up were excluded from the current study. The original development-test (75%/25%) split was preserved in our analysis.

### 2.3 Uncertainty methods

Predictive uncertainty was estimated using three established UQ methods [17]: Monte Carlo (MC) dropout [5], deep ensembles [6], and test-time augmentation (TTA) [18].

For MC dropout, dropout layers remained active during inference, thus producing variation in model configuration and subsequently in prediction, and 50 stochastic forward passes were performed per independent validation case. For each endpoint, the dropout rate, out of [0.1, 0.2, 0.3, 0.4, or 0.5], yielding the highest AUC on the validation cohort was used.

Deep ensembles were constructed by training ten models with identical training/validation data with identical training/validation data (80%/20%) splits of the development cohort but different random weight initialisations.

TTA, in contrast, probes uncertainty by assessing prediction variability under plausible input perturbations. The same random augmentations used during training (e.g., rotations, flips; see Appendix A3) were applied to the independent validation cohort, and 50 augmented versions of each case were generated to obtain the corresponding prediction samples.

For all three methods, the mean predicted probability across all sampled predictions was used as the final prediction, and the uncertainty measures applied across these samples were used to quantify patient-level uncertainty.

### 2.4 Uncertainty measures

For each uncertainty method, three commonly used uncertainty measures were compared: entropy, variance, and mutual information (MI). For entropy, the binary entropy function is used. Variance measures the dispersion of predicted probabilities across the sampled predictions. Finally, MI [5] captures the information gain about model parameters from the predictions, and is often interpreted as a measure of epistemic uncertainty. Formulas for these measures are provided in Appendix B.

### 2.5 Evaluation metrics

All models were evaluated on an independent validation cohort. The discriminative performance was assessed using AUC and accuracy. The calibration of the model’s predictions, and the uncertainty estimates, against the prediction accuracy was assessed quantitatively using the Adaptive Calibration Error metric (ACE, see Appendix C for definition) [19] and visually through calibration plots. Calibration bins were defined to contain at least 25 patients each, while a classification threshold of 0.5 was used for all accuracy evaluations. For the calibration assessments, uncertainty values measured by either variance or MI were normalised using min–max scaling, while entropy is already bounded between 0 and 1.

The association between predictive uncertainty and model performance was assesed using a sparsification analysis: for each uncertainty method–measure combination, patients in the independent validation cohort were ranked by descending uncertainty, and the most uncertain cases were iteratively removed while recalculating the AUC after each removal step. A well-calibrated uncertainty estimate should yield an increasing AUC as uncertain predictions are excluded, indicating effective discrimination between reliable and unreliable predictions.

### 2.6 Training set size experiment

To assess the relevance of dataset size on the uncertainty values, all of the UQ models were retrained using different sizes of training cohorts while maintaining a constant validation and independent validation cohort. Larger training cohorts are expected to lead to lower epistemic uncertainty values [20]. The training cohort was incrementally expanded by repeatedly sampling N additional patients from the original training cohort: first N patients were sampled to train an initial model, then N more patients were added to create a training set of 2 N patients for a second model, then N more for 3 N patients, and so on. This process continued until fewer than N unsampled patients remained in the original training set. For the NTCP models, N = 100, and for the TCP models, N = 50. To ensure robustness, this entire procedure was repeated ten times per UQ model, with the mean and variance of the uncertainty values and evaluation metrics reported.

## 3 Results

The NTCP cohort included 1,205 patients, while the TCP cohort consisted of 340 patients. 22% of patients in the NTCP cohort had Grade ≥ 2 dysphagia at 6 months (19% pre-treatment), while 42% had moderate-to-severe xerostomia (10% pre-treatment). 24% of patients in the TCP cohort died, while 25% had a locoregional control failure, within 2 years. Both cohorts were each split into respective development and independent validation cohorts. There were no significant differences (Appendix D) between the development and independent validation sets within either the NTCP cohort or the TCP cohort for any of the characteristics shown in Table 1.

**Table 1:** Patient characteristics of the NTCP and TCP model cohorts. TNM staging version 7 was used for all cohorts.

	NTCP Cohort				TCP Cohort			
	Development		Independent Validation		Development		Independent Validation	
**Total**	964		241		255		85	
**Sex** (%)								
Male	732	(76)	183	(76)	171	(67)	63	(74)
Female	232	(24)	58	(24)	84	(33)	22	(26)
**Age** (mean (SD))	64.5	(9.8)	64.8	(10.9)	61.5	(8.5)	62.4	(7.8)

**Tumour site** (%)								
Oropharynx	366	(38)	88	(37)	255	(100)	85	(100)
Nasopharynx	79	(8)	21	(9)	0	(0)	0	(0)
Hypopharynx	53	(5)	12	(5)	0	(0)	0	(0)
Larynx	397	(41)	104	(43)	0	(0)	0	(0)
Oral Cavity	58	(6)	13	(5)	0	(0)	0	(0)
Other	11	(1)	3	(1)	0	(0)	0	(0)

**T-stage** (%)								
T0	1	(0)	1	(0)	0	(0)	0	(0)
Tis	5	(1)	1	(0)	0	(0)	0	(0)
T1	180	(19)	48	(20)	31	(12)	11	(19)
T2	276	(29)	78	(32)	59	(23)	20	(23)
T3	230	(24)	49	(27)	37	(15)	11	(13)
T4	271	(28)	64	(27)	128	(50)	38	(45)
Tx	1	(0)	0	(0)	0	(0)	0	(0)

**N-stage** (%)								
N0	440	(46)	111	(46)	43	(17)	20	(24)
N1	102	(11)	32	(13)	29	(11)	3	(4)
N2	394	(41)	89	(37)	173	(68)	59	(68)
N3	28	(3)	9	(4)	10	(4)	3	(4)

**Smoking** (%)								
Current smokers	410	(43)	108	(45)	126	(49)	38	(45)
Past smokers	416	(43)	102	(42)	100	(39)	35	(41)
Never smokers	138	(14)	32	(13)	29	(11)	12	(14)

**OPC P16 HPV** (%)								
Positive	165	(17)	45	(19)	111	(44)	30	(35)
Negative	172	(18)	36	(15)	119	(47)	47	(55)
Unknown	29	(3)	7	(3)	25	(9)	8	(10)
Other tumour sites	596	(62)	153	(63)	0	(0)	0	(0)

**WHO** (%)								
0–1	900	(93)	235	(98)	236	(93)	82	(96)
≥2	52	(6)	5	(2)	19	(7)	3	(4)
Unknown	12	(1)	1	(0)	0	(0)	0	(0)

**Treatment technique** (%)								
3D-CRT	62	(6)	11	(5)	0	(0)	0	(0)
IMRT	351	(36)	100	(41)	108	(42)	31	(36)
VMAT	326	(34)	86	(36)	96	(38)	33	(39)
IMPT	217	(23)	44	(18)	46	(18)	20	(24)
IMPT + VMAT	8	(1)	0	(0)	5	(2)	1	(1)

**Systemic treatment** (%)								
With	364	(38)	99	(41)	150	(59)	50	(59)
Without	600	(62)	142	(59)	105	(41)	35	(41)

Table 2 presents the AUCs and ACE scores (95% CI) for the baseline models and the three UQ methods across the four models on their independent validation sets. Overall, the UQ methods did not lead to severely altered AUCs or ACE scores compared with the baseline, except for TTA on the dysphagia model, which was significantly lower in AUC than the baseline model (see Appendix E for all p-values). For LRC, both MC dropout and TTA achieved higher AUCs than the baseline (0.62 and 0.64 vs. 0.58, respectively). The selected dropout rate for both NTCP models was 0.2, and 0.1 for both TCP models.

**Table 2:** AUCs and ACEs [95% CI] of the baseline model and models trained with uncertainty quantification methods, on the independent test set. * indicates significant differences in AUC relative to the baseline model, using the DeLong test (p < 0.05). Abbreviations: AUC, area under the receiver operating characteristic curve; ACE, adaptive calibration error; MC, Monte Carlo; TTA, test-time augmentation; LRC, locoregional control.

		Dysphagia	Xerostomia	Survival	LRC
AUC	Baseline	0.88 [0.83–0.92]	0.72 [0.65–0.78]	0.71 [0.57–0.84]	0.58 [0.42–0.73]
	MC dropout	0.86 [0.80–0.91]	0.71 [0.64–0.77]	0.74 [0.59–0.88]	0.62 [0.48–0.76]
	Deep ensemble	0.87 [0.81–0.92]	0.72 [0.65–0.78]	0.73 [0.61–0.85]	0.57 [0.43–0.72]
	TTA	0.80 [0.74–0.87]	0.71 [0.65–0.78]	0.72 [0.57–0.86]	0.64 [0.50–0.77]

ACE	Baseline	0.08 [0.06–0.14]	0.15 [0.11–0.22]	0.13 [0.08–0.23]	0.16 [0.12–0.28]
	MC dropout	0.07 [0.05–0.13]	0.18 [0.13–0.24]	0.10 [0.08–0.21]	0.21 [0.13–0.30]
	Deep ensemble	0.12 [0.08–0.16]	0.18 [0.14–0.26]	0.12 [0.08–0.24]	0.18 [0.14–0.30]
	TTA	0.10 [0.10–0.16]	0.08 [0.07–0.16]	0.10 [0.09–0.22]	0.22 [0.15–0.31]

**Fig. 2:**
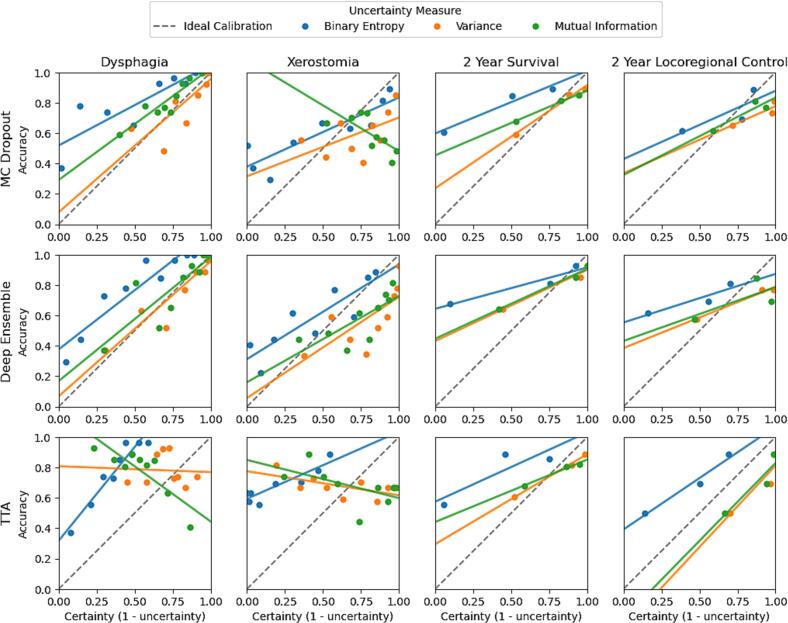
Calibration of uncertainty values against prediction accuracy. Abbreviations: MC: Monte Carlo, TTA: test time augmentation.

In Fig. 2, the calibration between the uncertainty values and the prediction accuracy is shown for each of the uncertainty methods and measures, for all four models. Therein, the certainty of the predictions almost always positively correlates with the accuracy of the predictions. The only exceptions to this are when MI and variance are used to measure the uncertainty of the NTCP models trained with TTA, and when MI is applied to the xerostomia MC dropout model. Across all endpoints, deep ensembles showed the best calibration between certainty and accuracy, compared to MC dropout and TTA. These trends were similar when the results on the independent validation cohort are stratified by primary tumour location (i.e. larynx and pharynx, see Appendix F), and when Youden’s J-index is used to define prediction thresholds during the calculations of accuracy (Appendix G).

**Fig. 3:**
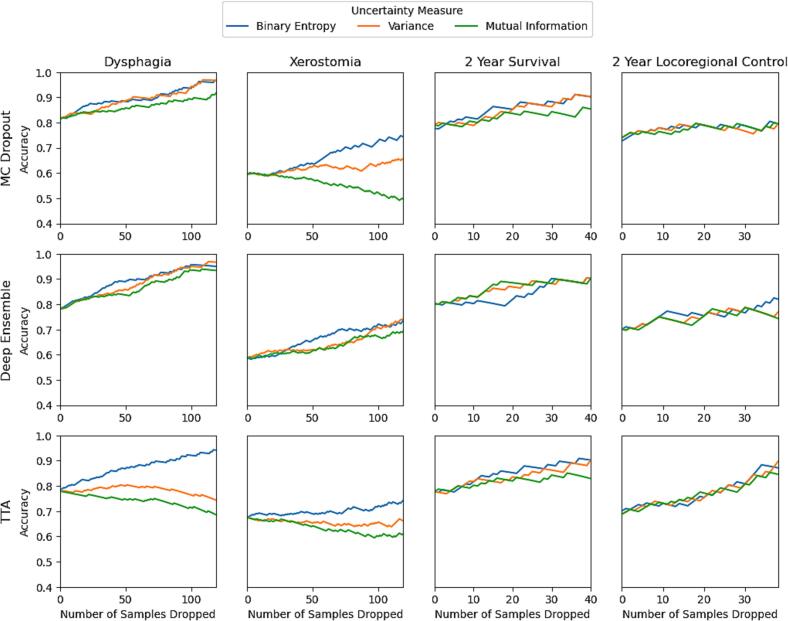
Sparsification plots of each uncertainty method-measure combination, for every endpoint. Abbreviations: UQ, uncertainty quantification; MC, Monte Carlo; TTA, test time augmentation.

Fig. 3 demonstrates the effect of iteratively removing the patient in the independent validation cohort with the most uncertain prediction on the accuracy of the models’ prediction on the aforementioned cohort, until half of this cohort has been removed. There, the accuracy of the dysphagia model increases from 0.8 to over 0.9 when the most ‘uncertain’ half of the predictions are removed, using MC dropout and deep ensembles. For all endpoints, these two uncertainty methods reflect higher accuracies on the more ‘certain’ half of the cohort. TTA also does so for 2-year survival and LRC, but presents mixed results on the NTCP models.

**Fig. 4:**
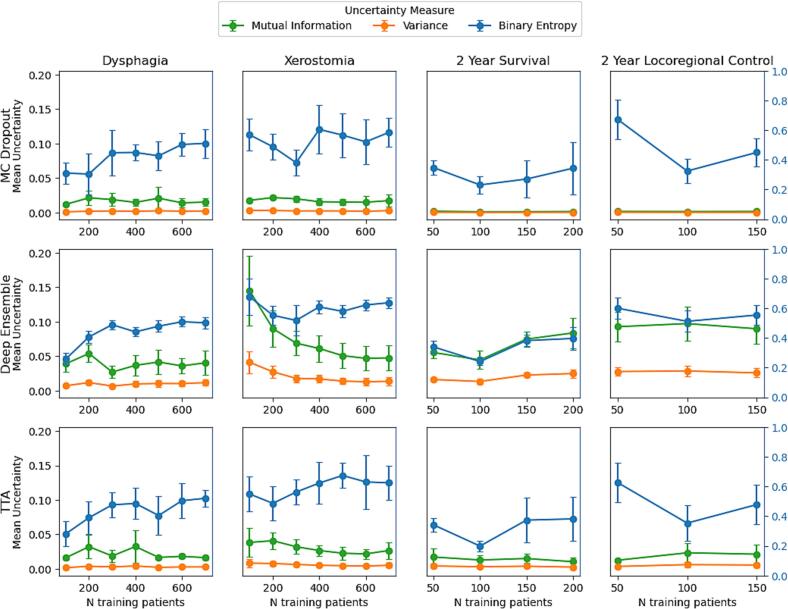
The mean and standard deviations of the uncertainty values over different sizes of training sets of each UQ method-measure combination, over ten iterations. As the values of binary entropy are on a much larger scale than mutual information and variance, the y-axis of binary entropy is plotted separately (on the right side, in blue). Abbreviations: MC, Monte Carlo; TTA, test time augmentation. (For interpretation of the references to colour in this figure legend, the reader is referred to the web version of this article.)

The effect of the training cohort size on the UQ values is shown in Fig. 4. For all models, the uncertainty of the predictions when using binary entropy either increases slightly as the training set increases in size, or remains relatively stable. The uncertainty values only decrease when using MI and variance together with the deep ensemble method on the xerostomia model. Further results for this experiment are shown in Appendix H, wherein the model’s AUC on the independent validation set does increase when the training cohort increases in size, while the calibration error of the uncertainty values against the model accuracy remains stable across all training cohort sizes.

## 4 Discussion

This study evaluated three established UQ methods and three uncertainty measures across four DL NTCP and TCP models on a large HNC cohort, demonstrating that UQ methods, such as MC dropout and deep ensembles, can be incorporated without predictive performance cost. Uncertainty estimates generally correlated with prediction accuracy, demonstrating that UQ methods could be a meaningful approach to present the reliability of a risk prediction.

However, the reliability of uncertainty estimates of outcome prediction models can vary greatly across different UQ methods and metrics. Deep ensembles and MC dropout consistently demonstrated strong calibration between uncertainty and prediction accuracy (Fig. 2), effectively capturing model uncertainty across diverse outcomes and patient cohorts. Their robust performance stems from their ability to probe different aspects of the learned model; ensembles by training multiple independent models that can disagree, and MC dropout by introducing controlled randomness during prediction [5], [6]. In contrast, TTA showed highly variable performance depending on the outcome and uncertainty measure, including significantly lower AUCs for dysphagia prediction compared to the baseline model. This likely stems from the fundamental challenge of defining meaningful augmentation policies for multimodal NTCP/TCP models that jointly process 3D CT/PET images, dose distributions, organ/GTV contours, and clinical tabular features. For example, gaussian noise injection may reasonably simulate sensor noise in CT inputs but could be a relatively less realistic augmentation for dose distributions and cannot be meaningfully applied to clinical features. Without modality-aware augmentation schemes, TTA risks injecting artefactual variation rather than capturing genuine uncertainty.

Differences were also observed in how well the three uncertainty measures captured prediction uncertainty. Among them, entropy and variance most consistently showed a positive correlation between uncertainty and accuracy, even though entropy tended to overestimate uncertainty. In contrast, MI was the most unstable measure, showing noisy or erratic values for several UQ methods. This aligns with prior findings that MI can be numerically unstable in binary classification because it is computed as a difference of two small entropies, amplifying Monte Carlo sampling noise [5], [21], [22], suggesting MI is not suitable for use in DL outcome prediction models.

UQ in DL–based outcome prediction faces fundamentally different challenges than tasks such as auto-segmentation. Unlike segmentation tasks with dense voxel-wise supervision, NTCP/TCP models predict sparse patient-level outcomes, often months or years after treatment, with labels that can be missing, censored, or prone to patient- or physician-reported variability [23]. This inherent difficulty is reflected in the elevated entropy values observed across the models and the slight increase in uncertainty (binary entropy) as training cohort size grows (Fig. 4). We hypothesize that this increase occurs because the models underestimate the true aleatoric uncertainty when the training sample size is too small, and that this uncertainty greatly outweighed epistemic uncertainty. Changes in treatment techniques and protocols over time can further exacerbate cohort heterogeneity; consequently, adding newly-collected patient data not only increases the size of the training cohort but also its diversity, which can further complicate the modelling task. In this study, much greater variation in the behaviour of different UQ methods was observed than in van Aalst et al. [24] observed in their evaluation of UQ methods for organ-of-interest segmentation models for HNC, highlighting that UQ methods validated in segmentation cannot be assumed to generalize directly to patient-level outcome prediction and emphasizing the need for careful validation in NTCP and TCP models.

A key limitation of this study is that the NTCP and TCP cohorts were derived from a single institution, and the UQ methods were not evaluated on external datasets. Consequently, the relationships between uncertainty and model error observed here may not fully generalise to external cohorts with different planning protocols, contouring practices, or patient populations. Additionally, min–max scaling was applied to compare entropy, variance, and mutual information across models because these metrics operate on different scales. As these scaling parameters were derived from the independent validation cohort, the calibration analyses may be partially conditioned on this distribution and should therefore be interpreted as a comparative assessment of UQ methods and measures rather than a deployment-ready calibration strategy. Moreover, this normalisation approach precludes a direct comparison of the reliability of uncertainty estimates between models with differing predictive performances, as the rescaling removes absolute differences in uncertainty magnitude that may be informative in such comparisons. In clinical applications, predefined thresholds based on non-normalised uncertainty values could be used to flag unreliable predictions, for example by identifying a cutoff on a validation set where predictions achieve a desired accuracy (e.g., 90–95%, analogous to the sparsification plots in Fig. 3). Future work should therefore evaluate the reliability of UQ-based methodologies for detecting out-of-distribution predictions and supporting quality assurance of DL outcome prediction models, both within and between different institutions.

Another limitation is that we binarised the TCP endpoints used by Ma et al. [16] to apply probabilistic UQ methods, as these methods and measures cannot be directly translated to the original event-based endpoints. We also did not evaluate conformal prediction, which produces prediction sets rather than probabilities; this study focused on probability-based UQ methods as these align better with how NTCP values are used clinically to guide treatment decisions [25].

While our results demonstrate positive correlations between uncertainty and model error, future work can explore how these uncertainty estimates could be optimally presented and applied in real-world clinical decision support workflows. As highlighted by Wahid et al. [7], few studies have addressed the translation of UQ approaches into clinical practice. Nevertheless, our findings suggest practical clinical potential: across all NTCP and TCP endpoints, MC dropout and deep ensembles showed positive correlations between uncertainty values and model errors, indicating that uncertainty provides useful information about prediction reliability. The sparsification experiments (Fig. 3) demonstrated that removing the most uncertain predictions substantially improved overall accuracy, suggesting opportunities for selective prediction workflows where clinicians prioritize high-confidence predictions while reviewing uncertain cases. Methods for integrating these uncertainties into decision-making through intuitive visualizations—such as visual dashboards, confidence scores, or risk ranges— will be critical for the safe and effective integration of UQ-integrated DL models into personalised radiotherapy.

This study demonstrates that UQ using MC dropout and deep ensembles can provide meaningful information about model confidence in NTCP and TCP prediction, with higher uncertainty generally associated with greater prediction error. Importantly, applying these two UQ methods to existing DL outcome prediction models did generally not diminish model performance. This study also showed that UQ in outcome modelling is more challenging than in auto-segmentation due to sparse, noisy, patient-level endpoints and multimodal inputs, leading to greater variability across methods. These findings highlight both the potential utility of UQ for selective prediction in clinical workflows and the need for careful validation before widespread implementation in prediction strategies.

## Funding statement

This work was funded by KWF 10.13039/501100004622Dutch Cancer Society via a Young Investigator Grant (KWF-13529). Moreover, L.V. van Dijk, received/receives salary support related to this project from 10.13039/501100003246NWO 10.13039/501100001826ZonMw via the VENI (10.13039/501100003246NWO-09150162010173).

## CRediT authorship contribution statement

**D.C. MacRae:** Writing – original draft, Visualization, Software, Methodology, Investigation, Formal analysis, Data curation, Conceptualization. **L. van der Hoek:** Writing – review & editing, Methodology, Investigation, Software, Data curation. **J.E. van Aalst:** Writing – review & editing, Methodology, Investigation. **S.P.M. de Vette:** Writing – review & editing, Data curation. **R. van der Wal:** Software. **H. Neh:** Data curation. **B. Ma:** Data curation. **N.M. Sijtsema:** Writing – review & editing, Supervision, Methodology. **M.A. Valdenegro-Toro:** Writing – review & editing, Supervision, Methodology. **P.M.A. van Ooijen:** Writing – review & editing, Supervision, Methodology. **L.V. van Dijk:** Writing – review & editing, Supervision, Funding acquisition, Conceptualization.

## Declaration of competing interest

The authors declare that they have no known competing financial interests or personal relationships that could have appeared to influence the work reported in this paper.

## Appendix A Supplementary data

Supplementary Data 1 Supplementary data including model training procedures, uncertainty measure equations, and results of ablation experiments.
