# Supplementary material for: An evaluation of uncertainty quantification methods and measures for deep learning outcome prediction models in head and neck cancer radiotherapy
Source: Phys Imaging Radiat Oncol. 2026 Apr 26;39:100978. doi: 10.1016/j.phro.2026.100978 (PMC13144595; doi:10.1016/j.phro.2026.100978)
Supplement: Supplementary Data 1 — Supplementary data including model training procedures, uncertainty measure equations, and results of ablation experiments. [file mmc1.pdf]

Appendix A: DL models

A.1 DL model architectures

The NTCP models [1], [2] both consist of 3D ResNet backbones and a set of linear layers, the latter of which combine the image features with clinical features (tabular data) before outputting the prediction. They accept CT, dose distribution, and organ-of-interest images, all 3D, as input to the ResNet backbone. These images were resampled to a resolution of $2 \times 2 \times 2\ \text{mm}^3$, and cropped to a model input dimension of $96 \times 96 \times 96$, using the same guidelines as in the original publications.

The TCP models are TransRP model architectures [3]; a combination of a DenseNet image encoder, a vision transformer (ViT), and a set of linear layers. In contrast to the NTCP models, the TCP models utilise the ViT to combine the image features with the clinical features (rather than do so using the linear layers). The image inputs also differ slightly from the NTCP models, rather than the dose distribution, the 3D PET scan is used. The resolution and dimensions of the image inputs are the same as the NTCP models and the original publication.

A.2 DL model training procedure

Our experiments are implemented using the PR3DICTOR framework (https://github.com/DLinRadiotherapyUMCG/PR3DICTR) for deep learning outcome prediction modelling. The code for the experiments themselves is provided in GitHub: https://github.com/daniel-macrae/RT_outcome_prediction_UQ_approaches_evaluation. Models were trained using Python 3.9 and PyTorch 2.6, using an Intel Xeon Platinum 8562Y+ CPU and an Nvidia L40S GPU.

All models were trained for a maximum of 200 epochs, or until the validation loss did not improve for more than 10 consecutive epochs. The (training) hyperparameters of each model were kept identical to their respective publications, with the exception that the output layer of the TCP models was adapted to output only a single predicted probability (using the sigmoid function), rather than event-based endpoints. Accordingly, the loss function for the TCP models was changed from negative log-likelihood to the binary cross entropy function.

A.3 Training- and Test-Time Augmentations

During model training, we applied randomized image augmentations to increase dataset diversity and improve generalisability. Test-time augmentation (TTA) uses the same augmentation pipeline. All spatial augmentations were applied jointly across the CT, dose, and contour channels to preserve alignment. A fixed random seed was used for reproducibility, and augmentations were sampled independently for each forward pass. For each patient—whether during training (per epoch) or during a TTA pass—each

augmentation listed in Table A.1 was independently applied with a probability of 50%. The ranges and intensities of the augmentations are provided in Table A.1.

Table A.1: Image augmentation parameters used during the training of the NTCP and TCP models. The same parameters were applied during test time augmentation.

**Augmentation method**	**Strength**
Flipping	Flip across the y-axis (left-right flip)
Affine transformation	Max translation of 6 voxels
Gaussian noise	Mean: 0, standard deviation: 0.01
Random cropping	Crops the image arrays to a new centre point (max 4 voxels away from the old centre point)
Rotation	0-15 degrees rotation within the x-axis (transverse plane)

Appendix B: Uncertainty measures

The formulas for the three uncertainty measures evaluated in this study (binary entropy, variance, and mutual information) are shown below.

Considering an outcome prediction task with one class, we first sampled T samples using a sampling method (Monte Carlo dropout, deep ensemble, or test time augmentation). For each input x, we obtained a set of T predictions P where $P = [p_1(y = 1 \mid x)$, $p_2(y = 1 \mid x)$, … , $p_T(y = 1 \mid x)]$ and each $(p_t(y = 1 \mid x) \in [0,1])$ represents the predicted probability for this outcome, from the t-th sampled prediction.

The mean predicted probability is computed as:

$$\bar{p}(y = 1 \mid x) = \frac{1}{T}\sum_{t=1}^{T} p_t(y = 1 \mid x)$$

1. Binary Entropy (Predictive Entropy)

The predictive entropy measures the uncertainty in the average prediction. It is defined below for any prediction p, where p is shorthand for $\mathrm{p}(y = 1 \mid x)$. When binary entropy is described in the main manuscript as the uncertainty measure, then this function is applied to the mean prediction $\bar{p}$.

$$H[p] = -p\log_2(p) - (1 - p)\log_2(1 - p)$$

Note: The entropy ranges from 0 (completely certain) to 1 (maximum uncertainty when $(p = 0.5)$*). By convention,* $(0\log(0) = 0)$*.*

2. Variance

The variance quantifies the spread of predictions across different dropout samples (i.e. the set of predictions P), measuring the disagreement between individual forward passes:

$$\mathrm{Var}[P] = \frac{1}{T}\sum_{t=1}^{T}(p_t - \bar{p})^2$$

Note: Higher variance indicates greater disagreement between different dropout masks, suggesting epistemic uncertainty.

3. Mutual Information

Mutual information decomposes the total uncertainty into aleatoric (irreducible data uncertainty) and epistemic (model uncertainty) components:

$$I_\theta[P] = H[\bar{p}] - E_\theta\big[H[P]\big]$$

where:

- $H[\bar{p}]$ is the predictive entropy (total uncertainty) of the predictions, as defined above using the binary entropy of the mean prediction $\bar{p}$.
- $E_\theta\left[H[P]\right]$ is the expected entropy (average entropy) of all of the individual predictions, representing aleatoric uncertainty. The expected conditional entropy is computed as:

$$E_\theta\left[H[P]\right] = \frac{1}{T}\sum_{t=1}^{T} H[p_t]$$

Note: Mutual information captures epistemic uncertainty—the amount by which knowing the true model parameters would reduce our uncertainty about the prediction. Higher MI indicates the model is uncertain about which parameters to use.

Appendix C: Adaptive calibration error (ACE) metric

The Adaptive Calibration Error (ACE) is a bin-based metric that quantifies the discrepancy between predicted probabilities and observed outcome frequencies while ensuring approximately equal sample sizes per bin.

Let $\{(p_i, y_i)\}_{i=1}^{N}$ denote the set of predicted probabilities $p_i \in [0,1]$ and corresponding binary outcomes $y_i \in \{0,1\}$. The predictions are sorted in ascending order and partitioned into B bins $\{S_b\}_{b=1}^{B}$of equal size (i.e., $| S_b | \approx N/B$).

For each bin b, the average predicted probability ($\bar{p}_b$) and empirical outcome frequency ($\bar{y}_b$) are computed as

$$\bar{p}_b = \frac{1}{| S_b |} \sum_{i \in S_b} p_i$$

$$\bar{y}_b = \frac{1}{| S_b |} \sum_{i \in S_b} y_i \, .$$

The ACE is then defined as

$$\text{ACE} = \frac{1}{B} \sum_{b=1}^{B} | \bar{p}_b - \bar{y}_b | \, .$$

In contrast to the Expected Calibration Error (ECE), which uses fixed-width probability bins and weights each bin by its sample size, ACE employs adaptive bins with equal sample counts, reducing sensitivity to binning choices and improving robustness in regions with sparse predictions.

Appendix D: Patient cohort

Table D.1 contains the p-values for the evaluations of the differences between the development and independent validation sets, for both the NTCP and TCP datasets. They reflect the patient cohort characteristics presented in Table 1 of the manuscript. A chi-squared test was used for all characteristics, except for the 'age' category, for which a Mann-Whitney U test was used.

Table D.1: *p-values of significance tests between the development and test sets, for each of the two cohorts used in this study*.

	**NTCP Cohort**	**TCP Cohort**
**Sex**	1.0	0.294
**Age**	0.538	0.461
**Tumour site** (%)	0.939	1.0
**T-stage**	0.718	0.571
**N-stage**	0.485	0.108
**Smoking**	0.757	0.686
**OPC P16 HPV**	0.681	0.445
**WHO**	0.232	0.717
**Treatment technique**	0.181	0.656
**Systemic treatment**	0.382	0.586
**CT with contrast**	0.738	0.209
**CT with metal artefact**	0.995	0.214

Appendix E: Significance values model performance

Table E.1: p-values of the DeLong tests comparing the AUCs of each of the UQ models to the baseline model, for each endpoint. Underlined values indicate significance (p<0.05)

	**Dysphagia**	**Xerostomia**	**Survival**	**LRC**
**MC dropout**	0.108	0.578	0.435	0.472
**Deep ensemble**	0.453	0.765	0.415	0.838
**TTA**	0.004	0.927	0.765	0.297

Appendix F: Results within different primary tumour sites

Figures F.1 and F.2 present the calibration between the certainty values and accuracies of the predictions, when the model results on the independent validation cohort is stratified by primary tumour locations (pharynx and larynx). Comparisons between other relevant sub-populations, such as photon and proton treatments, were not possible due to limited samples sizes.

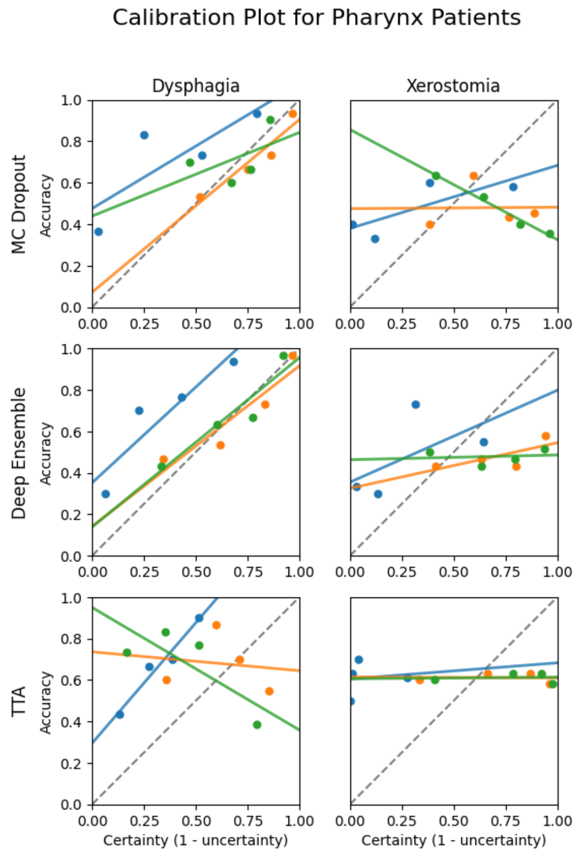

Figure F.1: *Calibration of uncertainty values against prediction accuracy, for patients in the independent validation cohort with primary tumours in the pharynx region. Abbreviations: MC: Monte Carlo, TTA: test time augmentation.*

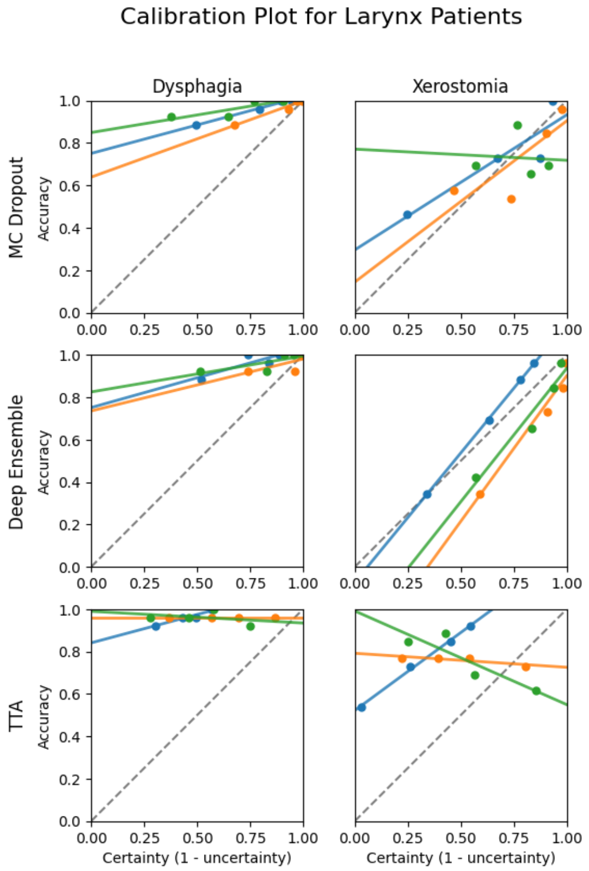

Figure F.2: *Calibration of uncertainty values against prediction accuracy, for patients in the independent validation cohort with primary tumours in the larynx region. Abbreviations: MC: Monte Carlo, TTA: test time augmentation.*

Appendix G: Uncertainty-accuracy calibration with different prediction thresholds

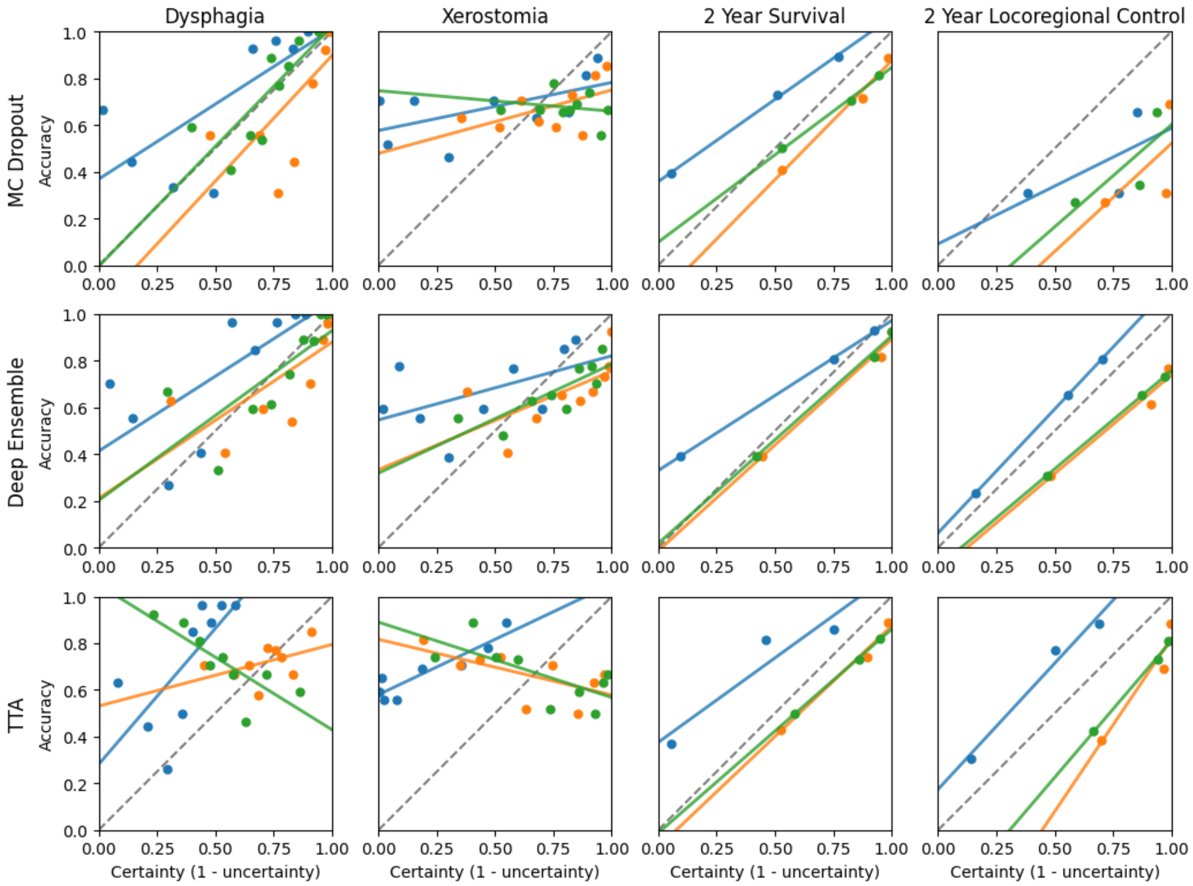

Figure G.1: Calibration between certainty (1-uncertainty) and accuracy of the models' predictions on the independent validation set, while using Youden's J-index to determine the threshold used during the calculation of the accuracy values.

Appendix H: Results of training data experiment

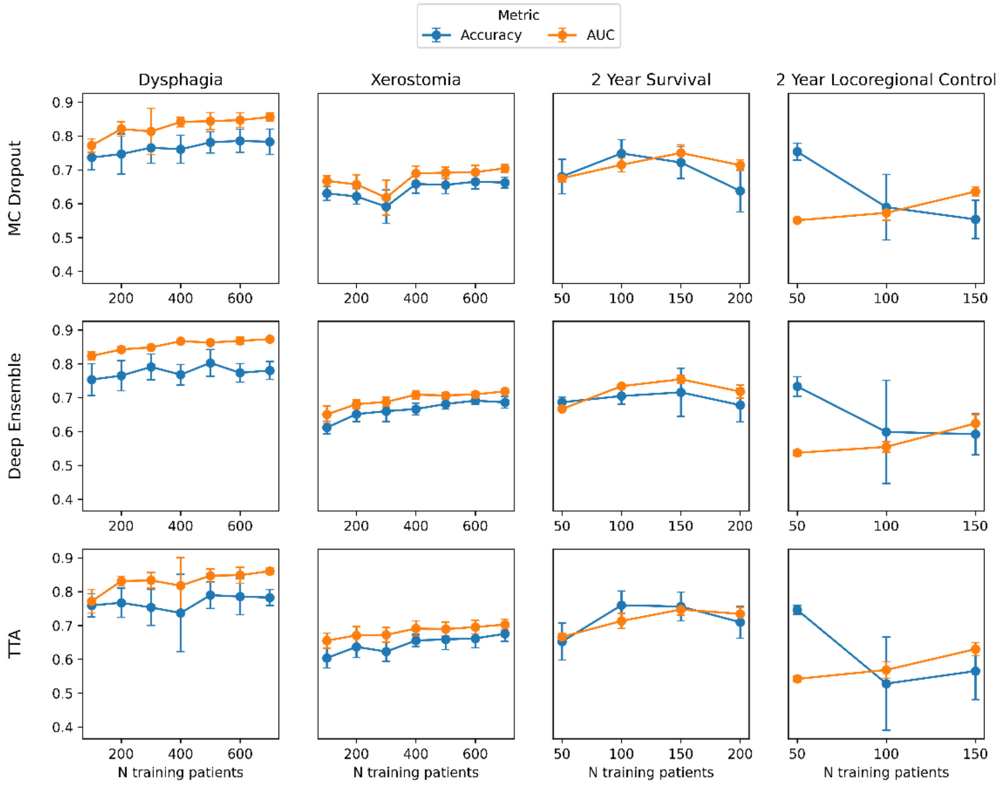

Figure H.1: Plot of AUC on the independent validation cohort, over different sizes of training sets, for each UQ method and model. The mean and standard deviation over ten iterations is shown.

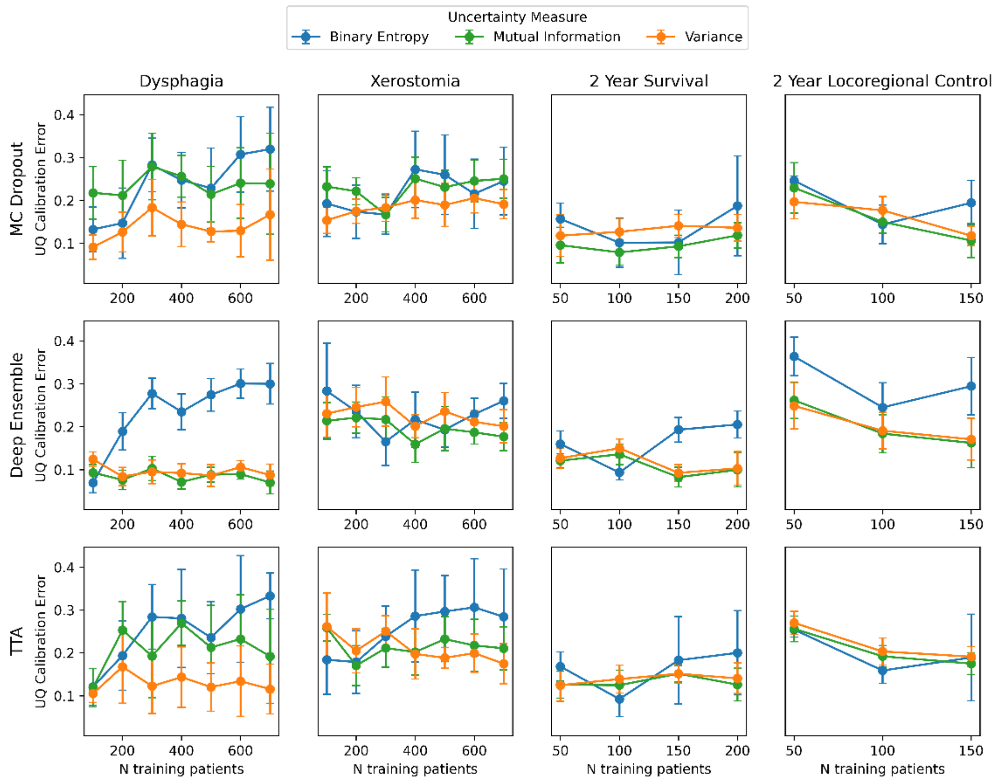

Figure G.2: Plot of ACE values, measuring the calibration error between the uncertainty values and accuracy on the independent validation cohort (lower values are better), across different sizes of training sets for each model and UQ method-metric combination. The mean and standard deviation over ten iterations is shown.
